# A Comparative Evaluation of the Postoperative Sensitivity of Bulk-Fill Versus Conventional Resin Class II Composite Restorations: A Prospective Observational Study

**DOI:** 10.7759/cureus.102366

**Published:** 2026-01-27

**Authors:** Ishita Agarwal, Lavanya Priya, Aishwarya Arya, Mary Grace, Ola Mohamed Sakr, Piyushi Tiwari, Rahul Tiwari, Seema Gupta

**Affiliations:** 1 Department of Conservative Dentistry and Endodontics, Uttaranchal Dental and Medical Research Institute, Dehradun, IND; 2 Department of Conservative Dentistry and Endodontics, Asian Institute of Medicine, Science and Technology (AIMST) University, Bedong, MYS; 3 Department of Conservative Dentistry and Endodontics, Vyas Dental College and Hospital, Jodhpur, IND; 4 Department of Pediatric Dentistry, Varas Dental Care, Chennai, IND; 5 Department of Conservative Dental Sciences, College of Dentistry, Qassim University, Burydah, SAU; 6 Department of Conservative Dentistry and Endodontics, RKDF Dental College and Research Centre, Bhopal, IND; 7 Department of Oral and Maxillofacial Surgery, RKDF Dental College and Research Centre, Bhopal, IND; 8 Department of Orthodontics, Kothiwal Dental College and Research Centre, Moradabad, IND

**Keywords:** composite resins, conventional, incremental, polymerization, postoperative, sensitivity

## Abstract

Introduction

Postoperative sensitivity can be a prevalent issue in class II composite restorations, which may impact patient satisfaction levels. This prospective observational study aimed to compare the postoperative sensitivity of bulk-fill and conventional resin composites in class II restorations over one month.

Methods

Sixty-two patients with moderate-depth class II carious lesions in posterior teeth were consecutively enrolled. Restorations were performed using either a bulk-fill composite (Tetric EvoCeram Bulk Fill, Ivoclar Vivadent AG, Schaan, Liechtenstein) in a single 4-mm increment or a conventional composite (Filtek Supreme XTE, 3M ESPE, St. Paul, MN, USA) in 2-mm oblique increments, based on clinician judgment. The same universal adhesive and standardized operative protocol were used in both groups. Postoperative sensitivity was assessed using a Visual Analog Scale in response to thermal and occlusal stimuli at 24 h, one week, and one month. Clinical outcomes including restoration fractures, marginal gaps, and secondary caries were evaluated. Baseline characteristics were compared using independent t-tests and chi-square tests. Sensitivity scores were analyzed using Mann-Whitney U tests and a mixed-effects model.

Results

Both groups were comparable in terms of age, cavity depth, sex, and tooth type. Median sensitivity scores were similar at 24 h and one month, but significantly lower in the bulk-fill group at one week. The mixed-effects model revealed a significant main effect of material, with overall lower sensitivity in the bulk-fill group and a significant reduction over time in both groups without an interaction effect. The quantitative analysis indicated a reduced incidence of restoration fractures, marginal discrepancies, and secondary caries within the bulk-fill cohort; however, these differences did not reach statistical significance.

Conclusion

Bulk-fill composite restorations exhibited a markedly reduced overall postoperative sensitivity in comparison to traditional composites in class II cavities, notably observable at the one-week mark, while also demonstrating similar short-term clinical efficacy.

## Introduction

Dental caries remain one of the most widespread chronic diseases worldwide, affecting billions of individuals and frequently requiring restorative procedures to protect tooth structure and maintain function [1,2]. In posterior teeth, class II cavities that involve the proximal surfaces and commonly include occlusal regions create distinct challenges because of their difficult access, necessitating materials that deliver strength, visual appeal, and a minimally invasive approach [1,2].

In recent decades, resin-based composites have significantly changed restorative dentistry by replacing conventional amalgam restorations in various clinical cases [1,3]. These materials provide better esthetics, effective bonding to dental tissues, and a mercury-free composition, which better meets patients' preferences for restorations that appear natural [4]. However, certain drawbacks continue to exist, especially in deeper preparations, where shrinkage during polymerization can produce stresses along the tooth-restoration boundary, possibly resulting in microleakage, recurrent caries, or pulpal irritation [5].

Standard resin composites, which have long been the primary choice, must be applied in successive layers no thicker than 2 mm to allow proper light curing of the entire material. This incremental method helps limit the shrinkage volume but lengthens the treatment duration, increases the possibility of voids or contamination across layers, and requires high levels of clinical expertise [6]. Despite progress in formulations, including nanofillers to enhance physical properties, issues such as postoperative sensitivity are still frequently reported, typically presenting as pain from temperature changes or biting forces due to inadequate polymerization, bacterial ingress, or fluid dynamics in dentinal tubules [7,8].

To address these limitations, bulk-fill composites have been developed as a newer option, permitting placement in a single increment of up to 4-5 mm. They feature altered resin systems, additives to alleviate stress, and improved light-activation components for a greater depth of cure and reduced shrinkage forces [9,10]. The advantages of this technique include easier handling, shorter operative time, and superior flow into the complex posterior areas. Preliminary research has shown similar survival rates and edge sealing compared to traditional composites, although results regarding patient-reported outcomes, such as reduced postoperative sensitivity, potentially from lower stress at the interfaces, are not entirely consistent [9,10].

Postoperative sensitivity following composite placement can occur in as many as 30% of cases, directly affecting patient comfort and overall treatment acceptance. The important contributing aspects include the depth of the cavity, application of base liners, and inherent material traits [7,10]. Nonetheless, head-to-head evaluations of bulk-fill and conventional composites, specifically in class II restorations, are still scarce, particularly those involving extended prospective follow-ups.

This study aimed to compare postoperative sensitivity in class II restorations using bulk-fill composites versus conventional resin composites. The primary objective of this prospective observational study was to compare postoperative sensitivity between class II restorations placed using bulk-fill resin composite and those restored with conventional incremental resin composite at 24 hours, one week, and one month postoperatively using a visual analog scale. The secondary objectives were to evaluate the change in postoperative sensitivity over time within each restorative group, to assess short-term clinical performance outcomes including restoration fractures, marginal gaps, and secondary caries, and to explore the influence of patient- and cavity-related factors such as age, cavity depth, and tooth type on postoperative sensitivity.

## Materials and methods

Study design

This prospective observational cohort study was conducted at the Department of Conservative Dentistry and Endodontics, RKDF Dental College and Research Centre, Bhopal, India, from December 2024 to June 2025. Participants were consecutively enrolled and followed prospectively after receiving standard restorative treatment, with the choice of material determined by the treating clinician based on clinical judgment (cavity depth, operator preference, or time constraints). Two cohorts were observed: one was restored with a bulk-fill composite, and the other with a conventional resin composite. Ethical authorization was secured from the institutional review board before the initiation of the research endeavor (RKDF/DC/2024/S92 dated November 17, 2024). All methodologies adhered to the principles outlined in the Declaration of Helsinki, and informed consent was duly acquired from every participant involved.

Participants

The required sample size was determined a priori using G*Power software (version 3.1.9.2; Heinrich Heine University, Düsseldorf, Germany) for a two-proportion comparison. The analysis assumed a baseline sensitivity of 25% for the conventional composite group and an expected sensitivity of 40% for the comparison group [11]. To detect this difference with a two-sided alpha error of 5% and statistical power of 80%, the calculation indicated a minimum of 31 participants per group was necessary. Therefore, a total sample size of 62 (31 bulk-fill and 31 conventional composites) was required for this study.

A total of 62 participants were consecutively recruited from patients seeking routine dental care, aged 18-65 years, who presented with at least one class II carious lesion in a posterior tooth (premolar or molar) requiring restoration. Patients were screened during the initial examinations using clinical and radiographic assessments to confirm moderate-depth caries without pulpal involvement. Demographic data including age, sex, oral hygiene habits, medical history, and reasons for material selection were recorded to assess potential confounding factors.

Inclusion and exclusion criteria

Inclusion criteria included healthy adults with vital posterior teeth exhibiting class II cavities (proximal and occlusal involvement) of moderate depth, as determined by bitewing radiographs showing caries extending up to the middle third of the dentin but not involving the pulp. The teeth responded positively to vitality tests and were free from periodontal disease or cracks. Patients with allergies to resin-based materials, pregnant or lactating women, those with severe bruxism or parafunctional habits, teeth with previous restorations or endodontic treatment, and individuals unable to attend follow-up visits were excluded. Patients taking medications affecting pain perception, such as chronic analgesics, were also excluded to minimize bias in sensitivity reporting. Only one restoration per participant was included in the analysis to ensure the independence of observations.

Cohort allocation and blinding

Cohorts were defined post-treatment based on the restorative material used in routine care, without randomization. The material selection was performed at the discretion of the treating clinician. Evaluators assessing postoperative sensitivity were independent and blinded to the material used to reduce detection bias. Patients remained unaware of specific material differences where possible, although complete blinding was limited owing to observable procedural variations.

Materials and instruments

The bulk-fill composite used was the Tetric EvoCeram Bulk Fill (Ivoclar Vivadent AG, Schaan, Liechtenstein), a posterior restorative material with enhanced depth-of-cure properties. The conventional resin composite used was Filtek Supreme XTE (3M ESPE, St. Paul, MN, USA), a nanofilled universal composite applied in incremental layers. Etching was performed using 35% phosphoric acid gel (Ultra-Etch, Ultradent Products, Inc., South Jordan, UT, USA). Bonding involved Scotchbond Universal Adhesive (3M ESPE, St. Paul, MN, USA), which is a self-etch universal adhesive. Isolation was achieved using a rubber dam system (Hygenic Dental Dam, Coltene/Whaledent Inc., Altstätten, Switzerland). Cavity preparation was performed using high-speed handpieces with diamond burs (Kerr Dental, Brea, California, USA) under water coolant, and finishing was performed using Sof-Lex discs (3M ESPE, St. Paul, MN, USA). Curing was performed with a light-emitting diode (LED) light unit (Bluephase Style, Ivoclar Vivadent AG, Schaan, Liechtenstein) at an intensity of 1200 mW/cm² [8].

Operative procedure

All restorative procedures were carried out by a single experienced clinician (endodontist with more than five years of clinical experience), which helped minimize operator-related variability. Local anesthesia (2% lidocaine with 1:100,000 epinephrine) was administered as needed. Rubber dam isolation was applied, followed by cavity preparation using conservative principles to remove caries and create bevels on enamel margins. For both cohorts, the cavity was etched for 15 s on dentin and 30 s on enamel with ultra-etch, rinsed, and gently air-dried. Scotchbond Universal Adhesive was actively applied for 20 s, air-thinned, and light-cured for 10 s. In the bulk-fill cohort, the Tetric EvoCeram Bulk Fill was placed in a single 4-mm increment and cured for 20 s. In the conventional cohort, Filtek Supreme XTE was placed in 2-mm oblique increments, each cured for 20 s. Occlusal anatomy was contoured pre-curing, followed by finishing and polishing with Sof-Lex discs under water spray. Immediate post-operative radiographs confirmed the restoration integrity [8]. For all class II restorations, a sectional matrix system with separation rings and wooden wedges was used to achieve proper proximal contact and anatomical contour, and the same matrix protocol was followed in both groups. Occlusal contacts were evaluated using 40-µm articulating paper, and necessary occlusal adjustments were performed before final polishing.

Assessment of postoperative sensitivity

Postoperative sensitivity was evaluated using a standardized protocol at specified intervals. Patients rated their sensitivity on a visual analog scale (VAS) from 0 (no pain) to 10 (severe pain) [12] in response to thermal stimuli (cold air from a syringe) and occlusal loading (biting on articulating paper). Clinical examination was performed for restoration fractures, marginal gaps, and secondary caries. Intra-examiner reliability was ensured using kappa statistics (>0.8). Any adverse events such as pulpitis requiring intervention were recorded and managed accordingly.

Statistical analysis

Statistical analysis was performed using Statistical Package for Social Sciences (SPSS) Statistics for Windows, version 27.0 (IBM Corp., Armonk, NY, USA). Data were initially screened for completeness and distributional assumptions. Continuous variables were assessed for normality using visual inspection of histograms and the Shapiro-Wilk test. Continuous variables with an approximately normal distribution (age and cavity depth) are presented as mean±standard deviation (SD) and were compared between the bulk-fill and conventional composite groups using the independent sample t-test. Categorical variables, including sex, tooth type, restoration fracture, marginal gap, and secondary caries, were presented as frequencies and percentages and compared between groups using the chi-square test of independence.

Postoperative sensitivity was measured using the VAS scores at 24 h, one week, and one month. As VAS scores did not follow a normal distribution, intergroup comparisons at each time point were performed using the Mann-Whitney U test, and the results were reported as medians with interquartile ranges. To evaluate changes in postoperative pain over time and assess the overall effect of restorative material while accounting for repeated measurements within participants, a mixed-effects model was applied. Time (24 h, one week, and one month) was included as a within-subject factor, and restorative material (bulk-fill or conventional composite) was included as a between-subject factor. The main effects of time and group as well as the interaction between time and group were examined. The effect sizes were reported using partial eta squared (η²).

## Results

A total of 62 class II restorations were evaluated, with 31 restorations in each study group (bulk-fill composite and conventional resin composite). All participants completed the scheduled follow-up assessments and no cases were excluded from the final analysis.

Baseline characteristics

The baseline characteristics of the study groups are summarized in Table 1. The mean age of participants in the bulk-fill group was 33.68±7.86 years, while that of the conventional composite group was 33.42±7.65 years. This difference was not statistically significant (p=0.896). Similarly, the mean cavity depth did not differ significantly between the bulk-fill group (3.39±0.65 mm) and the conventional group (3.45±0.74 mm; p=0.743), indicating baseline comparability between the groups.

**Table 1:** Baseline continuous characteristics of the study groups. Continuous variables are expressed as mean±standard deviation (SD). Independent-sample t-test was used for intergroup comparisons. CI: confidence interval. p>0.05 denotes no statistical significance.

Parameters	Group	95% Cl for mean	Mean±SD	t-value	p-value
Age (years)	Bulk-fill	30.79-36.56	33.68±7.86	0.131	0.896
	Conventional	30.61-36.23	33.42±7.65		
Cavity depth (mm)	Bulk-fill	3.15-3.62	3.39±0.65	-0.33	0.743
	Conventional	3.17-3.72	3.44±0.74		

Categorical characteristics and clinical outcomes

Categorical characteristics and clinical outcomes of the study groups are shown in Table 2. There were no statistically significant differences between the groups with respect to sex distribution (p=0.906) or restored tooth type (p=0.788). With regard to clinical outcomes, restoration fractures were observed in four cases (13%) in the bulk-fill group and nine cases (29%) in the conventional composite group; however, this difference was not statistically significant (p=0.119). Marginal gaps were detected in five restorations (16%) in the bulk-fill group and 11 restorations (35%) in the conventional group (p=0.080). Secondary caries were noted in three cases (10%) in the bulk-fill group and in seven cases (23%) in the conventional group (p=0.167). Although numerical differences favored the bulk-fill group, none of the outcomes showed statistically significant intergroup differences.

**Table 2:** Categorical characteristics and clinical outcomes of the study groups. Data are presented as frequency and percentage, Chi-square test of independence was applied, p>0.05 denotes no statistically significant difference between groups.

Variable	Category	Bulk-fill (n %)		Conventional (n %)		Chi stats (χ²)	p-value
		n	%	n	%		
Sex	Male	17	55%	16	52%	0.01	0.906
	Female	14	45%	15	48%		
Tooth type	Molar	20	65%	21	68%	0.07	0.788
	Premolar	11	35%	10	32%		
Restoration fracture	Yes	4	13%	9	29%	2.43	0.119
	No	27	87%	22	71%		
Marginal gap	Yes	5	16%	11	35%	3.03	0.080
	No	26	84%	20	65%		
Secondary caries	Yes	3	10%	7	23%	1.91	0.167
	No	28	90%	24	77%		

Intergroup comparison of postoperative sensitivity

The comparison of the postoperative sensitivity scores between the two groups at different time points is shown in Table 3. At 24 h post-restoration, both groups demonstrated a median VAS score of 0, and no statistically significant difference was observed between the bulk-fill and conventional groups (p=0.126). At the one-week follow-up, a statistically significant difference in postoperative sensitivity was detected between the groups (p=0.008), with the bulk-fill group demonstrating lower median VAS scores than the conventional composite group. At the one-month follow-up, the postoperative sensitivity scores decreased in both groups, and the intergroup difference was no longer statistically significant (p=0.094).

**Table 3:** Intergroup comparison of postoperative sensitivity (VAS scores) at different time points. Visual analog scale (VAS) scores are expressed as median and interquartile range (IQR), Mann-Whitney U test was used due to non-normal distribution of VAS scores, statistically significant at *p<0.05.

Timeline	Group	Median	Minimum	Maximum	Interquartile Range	U stats	p-value
24 hours	Bulk-fill	0	0	4	0	393.5	0.126
	Conventional	0	0	5	2		
1 week	Bulk-fill	0	0	7	3	308.5	0.008*
	Conventional	2	0	8	4		
1 month	Bulk-fill	0	0	10	5.5	371	0.094
	Conventional	2	0	10	6.5		

Longitudinal analysis of pain scores

The mixed-effects model analysis assessing changes in the postoperative pain scores over time is presented in Table 4. A significant main effect of time was observed (p=0.001), indicating a significant reduction in the pain scores across the follow-up periods. A significant main effect of restorative material was also found (p=0.043), with the bulk-fill composite group demonstrating overall lower postoperative pain scores than the conventional composite group. However, the interaction between time and group was not statistically significant (p=0.244), suggesting that the pattern of pain reduction over time was similar in both the groups.

**Table 4:** Mixed-effects model analysis of postoperative pain scores. Mixed-effects model evaluating main and interaction effects, df: degree of freedom, Partial eta squared (η²) indicates effect size, *p<0.05 denotes statistically significant value.

Effect	Sum of squares	df	Mean Square	F stats	p-value	η^2^
Time (24 h, 1 week, 1 month)	142.871	2	71.435	35.04	0.001*	0.102
Group (Bulk-fill vs. Conventional)	67.441	1	67.441	4.276	0.043*	0.048
Time x Group	5.817	2	2.909	1.427	0.244	0.004

Post-hoc analyses

Post-hoc pairwise comparisons across time points using the Bonferroni adjustment are shown in Table 5. Significant reductions in the VAS scores were observed from 24 h to 1 week (p=0.001) and from 24 h to 1 month (p=0.001). Additionally, a significant reduction was noted between the one-week and one-month follow-up periods (p=0.001). The post hoc comparisons between the study groups are summarized in Table 6. The bulk-fill group demonstrated significantly lower overall postoperative pain scores than the conventional composite group (mean difference=−1.204; p=0.043).

**Table 5:** Post-hoc pairwise comparisons of postoperative pain scores across time points (Bonferroni adjusted). *p=0.001 denotes statistically significant value.

Pairwise comparison	Mean difference	T stats	p-value	95% CI lower limit	95% CI upper limit
24 hours vs. 1 week	-1.145	-6.605	0.001*	-1.492	-0.798
24 hours vs. 1 month	-2.145	-6.166	0.001*	-2.841	-1.45
1 week vs. 1 month	-1	-4.584	0.001*	-1.436	-0.564

**Table 6:** Post-hoc comparison between study groups. Negative mean difference indicates lower pain scores in the bulk-fill group, *p<0.05 denotes statistically significant value.

Comparison	Mean difference	T stats	p-value
Bulk-fill vs. Conventional	-1.204	-2.068	0.043*

## Discussion

The findings of this prospective observational study highlight notable differences in postoperative sensitivity between class II restorations performed with bulk-fill and conventional resin composites while also revealing comparable clinical performance in other aspects. The observed lower sensitivity in the bulk-fill group, particularly evident at the one-week follow-up and sustained as an overall effect across assessment periods, aligns with the material's design advantages. The bulk-fill composite used in this study incorporates a high filler loading of approximately 79.5% by weight (including 62.5% inorganic fillers such as barium glass, ytterbium trifluoride, and mixed oxides, plus 17% prepolymerized "isofillers") and 61% by volume for standard fillers, to reduce the proportion of the reactive organic resin matrix (dimethacrylates accounting for approximately 19.7% by weight). This design minimizes the overall C=C to C-C conversion during polymerization, resulting in a lower volumetric shrinkage compared to materials with a lower filler content [13].

Additionally, a Raman spectroscopic study by Par et al. [14] showed that bulk-fill composites, including the Tetric EvoCeram Bulk Fill, exhibit a significant post-cure increase in the degree of conversion (11.3%-16.9%) within 24 h after light curing, which further improves the mechanical properties and interfacial adaptation over time. Tetric EvoCeram Bulk Fill has additional features, such as a patented shrinkage stress reliever (a pre-polymer that acts as a "chemical cushion" between filler particles) and enhanced photoinitiators, allowing deeper single-increment curing (up to 4 mm) while maintaining low shrinkage and elasticity. These elements collectively contribute to the observed benefits in postoperative sensitivity and adaptation and are likely to minimize dentinal fluid movement and pulpal irritation, which are key contributors to sensitivity, as opposed to the incremental layering in conventional composites, which may introduce interlayer voids or incomplete adaptation despite efforts to control contraction [15].

These results corroborate those of several prior investigations that have reported favorable outcomes for bulk-fill materials in terms of patient comfort. For instance, a randomized clinical trial by Al-Harbi et al. [16] evaluating bulk versus incremental placement of class II composites found no significant differences between the groups for marginal integrity. Similarly, a previous study comparing bulk-fill and conventional composites noted no significant difference in postoperative sensitivity incidence in posterior class I restorations [17]. Similarly, Costa et al. [18] concluded that the type of adhesive or filling technique (bulk/incremental) had no effect on postoperative sensitivity, and the overall risk of postoperative sensitivity was 20.3%.

The lack of difference at the 24-h and one-month marks, coupled with a uniform temporal decline in sensitivity for both groups, indicates that initial disparities may resolve as restorations stabilize. This pattern echoes previous studies that reported no significant differences in postoperative sensitivity between groups [11,17,18]. Previous systematic reviews and meta-analyses of clinical trials assessing bulk-fill versus conventional resins have concluded no overall superiority in sensitivity or clinical outcomes, with many trials showing equivalence due to advancements in adhesive systems that enhance bonding regardless of the placement method [9,10,19]. The absence of a significant time-material interaction in our analysis further supports the idea that both materials follow a similar trajectory of sensitivity resolution, possibly influenced by patient adaptation or resolution of transient inflammation.

Regarding secondary clinical outcomes, although not statistically significant, numerical trends favored the bulk-fill group, with fewer instances of restoration fractures, marginal gaps, and secondary caries. These observations may stem from the viscoelastic properties of the material, which promote better flow and adaptation to cavity walls, reducing microleakage pathways that could exacerbate long-term degradation. A previous study by Hardan et al. [20] similarly noted improved marginal integrity in bulk-fill restorations, with clinical evaluations demonstrating lower degradation risks than conventional composites. The comparable baseline demographics and cavity depths between the cohorts strengthen the validity of these comparisons, minimizing confounding from patient or lesion factors. The use of standardized VAS assessments and blinded evaluators further enhanced the reliability, aligning with protocols in prior sensitivity-focused trials.

The clinical implications of these findings are substantial for restorative dentistry. Bulk-fill composites appear to provide a viable alternative for class II restorations, potentially reducing short-term patient discomfort and procedural time without compromising early performance. This is particularly beneficial in busy clinical settings or for patients with limited tolerance for extended chair times, enhancing satisfaction and compliance. Clinicians may prioritize bulk-fill materials in moderate-depth posterior lesions, especially when aiming to minimize sensitivity risks, while ensuring optimal curing protocols to leverage their advantages.

The limitations of this study include the observational design, which relied on clinician discretion for material selection rather than randomization, thus introducing potential selection bias despite comparable baselines. The sample size, while meeting power calculations, may have limited the detection of subtle differences in rare events, such as fractures. Follow-up was restricted to one month for sensitivity, precluding insights into longer-term outcomes, such as wear or recurrent caries. Composite resin placement is a technique-sensitive procedure, and although efforts were made to minimize operator-related variability through protocol standardization and use of experienced clinicians, some degree of procedural bias cannot be completely eliminated. This limitation is inherent to clinical restorative studies and should be considered when interpreting the findings. Future randomized trials with extended monitoring and larger cohorts could address these gaps by incorporating advanced imaging techniques for subclinical assessments.

## Conclusions

This prospective observational study demonstrated that bulk-fill resin composite resulted in significantly lower overall postoperative sensitivity than conventional incremental resin composite in class II restorations, with the difference most pronounced at one-week post-placement. Although clinical outcomes, such as fractures, marginal gaps, and secondary caries, showed no statistically significant differences, numerical trends favored the bulk-fill group. These findings support the use of bulk-fill composites as a reliable and time-efficient alternative for posterior restorations, particularly in moderate-depth class II cavities, offering improved early patient comfort without compromising short-term performance.
